# Antimicrobial stewardship in the primary care setting: from dream to reality?

**DOI:** 10.1186/s12875-020-01191-0

**Published:** 2020-07-08

**Authors:** M. L. Avent, S. E. Cosgrove, E. G. Price-Haywood, M. L. van Driel

**Affiliations:** 1 Statewide Antimicrobial Stewardship Program, Queensland Health, Brisbane, Australia; 2 UQ Centre for Clinical Research (UQCCR), The University of Queensland, Brisbane, Australia; 3 Department of Medicine, Division of Infectious Diseases, Johns Hopkins University School of Medicine, Baltimore, MD USA; 4 Ochsner Health System, Center for Outcomes and Health Services Research, New Orleans, Louisiana USA; 5 Ochnser Clinical School, The University of Queensland, New Orleans, Louisiana USA; 6 Primary Care Clinical Unit, Faculty of Medicine, The University of Queensland, Brisbane, Australia

**Keywords:** Antimicrobial stewardship, Antibiotic stewardship, Antimicrobial, resistance; primary care; outpatient setting.

## Abstract

**Background:**

Clinicians who work in primary care are potentially the most influential healthcare professionals to address the problem of antibiotic resistance because this is where most antibiotics are prescribed. Despite a number of evidence based interventions targeting the management of community infections, the inappropriate antibiotic prescribing rates remain high.

**Discussion:**

The question is how can appropriate prescribing of antibiotics through the use of Antimicrobial Stewardship (AMS) programs be successfully implemented in primary care. We discuss that a top-down approach utilising a combination of strategies to ensure the sustainable implementation and uptake of AMS interventions in the community is necessary to support clinicians and ensure a robust implementation of AMS in primary care. Specifically, we recommend a national accreditation standard linked to the framework of *Core Elements of Outpatient Antibiotic Stewardship,* supported by resources to fund the implementation of AMS interventions that are connected to quality improvement initiatives. This article debates how this can be achieved.

**Summary:**

The paper highlights that in order to support the sustainable uptake of AMS programs in primary care, an approach similar to the hospital and post-acute care settings needs to be adopted, utilising a combination of behavioural and regulatory processes supported by sustainable funding. Without these strategies the problem of inappropriate antibiotic prescribing will not be adequately addressed in the community and the successful implementation and uptake of AMS programs will remain a dream.

## Background

The majority of antibiotics are prescribed outside of hospitals and the most common indication is acute respiratory tract infections (ARIs) [1, 2]. ARIs are mostly self-limiting and/or caused by viruses and therefore clinical guidelines generally do not recommend treatment with antibiotics. However, clinicians have high prescribing rates for these conditions [1] with only half of these prescriptions being concordant with guidelines and therefore considered appropriate [3]. Antibiotics are also associated with a number of complications ranging from rashes, diarrhea and severe allergic reactions [4]. In addition, they are a risk factor for *Clostridioides difficile* infection and community-acquired fluoroquinolone-resistant *E.coli* urinary tract infections [5, 6]. Therefore primary care clinicians are critical players in addressing the problem of inappropriate antibiotic prescribing [7].

Studies have shown that it is possible to reduce inappropriate antibiotic prescribing through behavioural interventions. Although initial findings are promising, limited information is available to determine whether they can be implemented and effective across a range of contexts in real world settings [8]. It is increasingly accepted that addressing inappropriate antibiotic prescribing requires a systems approach that has been described as Antimicrobial Stewardship (AMS) [9]. AMS is the coordinated set of actions designed to promote and increase the appropriate use of antimicrobials and is a key strategy to conserve the effectiveness of antibiotics into the future [10]. AMS strategies have mainly focused on the hospital and post-acute care settings. The success and sustainability of these programs have been attributed to linking Frameworks to national medication management and accreditation standards [11, 12]. Unfortunately, in the primary care setting adoption of AMS strategies has been neglected. Development and implementation of approaches to reduce overprescribing are urgently needed [11, 13]. .In acknowledgment of the lack of attention to AMS in clinical settings outside of hospitals and post-acute care settings, the Center for Disease Control (CDC) released a framework addressing the *Core Elements of Outpatient Antibiotic Stewardship* for outpatient facilities [14].

This paper argues that in order to improve the appropriate use of antibiotics in the community there is a need to support a bottom-up approach, i.e. the implementation and uptake of AMS interventions, by a top-down strategy, namely the application of a national accreditation standard linked to the Framework. Many evidence-based interventions have been trialled in research settings (Table 1). However, it is still unclear how they can be successfully implemented in a real-world setting. This article discusses how this can be achieved.

**Table 1:** Advantages and disadvantage of AMS interventions in the primary care setting

Core elements of AMS	AMS Interventions	Advantages	Disadvantages	Evidence of sustainability (study duration)
Commitment	1. Commitment poster in support of AMS [15] displayed in the waiting room and openly endorsed by the practice and its clinicians	Well received by the public. Provides standardised guidance & support for clinicians at a practice level. Low-cost, effective intervention.	Requires collaboration of clinicians at a practice level.	3 months
Action for practices	1. Clinical decision support [16–19]	Reduces inappropriate antibiotic prescribing.	Need for an Electronic Health Record system to support a Clinical Decision support tool. Low uptake can be a barrier to effectiveness.	6 to 18 months
	2. Back-up (delayed or ‘wait and see’) prescribing [20–24]	Decreases antibiotic use.	Antibiotics can be utilized for conditions other than the original presentation.	5 to 35 months
	3. Point of care tests [25–30]	Decreases diagnostic uncertainty. Supports non-prescription decisions. Decreases inappropriate antibiotic use for viral infections.	Lack of reimbursement for point of care tests. Over-reliance or under-reliance on diagnostic tests. Difficulty of incorporating tests into current practice work flow.	4 to 30 months
Tracking and reporting	1. Personalized audit and feedback to prescribers of antibiotic-prescribing rates in comparison to peers [16, 31–34] 2. Public reporting of antibiotic usage data [35]	Modifies prescribing behaviour. Reduces inappropriate prescribing.	Time consuming and increased resources are required for auditing process. Auditing process requires an Electronic Health Record system. Expertise required to validate and interpret data.	6 months to 3 years
Education and expertise	Patient education a. Shared decision making [36, 37]	Decreases antibiotic use. Improves patient satisfaction.	Time consuming for clinicians.	Limited length of observation
	Clinician education b. Communication training [26, 33, 38, 39] c. Peer academic detailing [40]	Promotes acceptance of AMS strategies. Tailored education such as communication training and peer academic training has proven to be effective in modifying prescriber behaviour. Effective with sustained benefits over time. Active, in-person education more effective than didactic education. Clinician education effectively supports other interventions.	Time consuming for clinicians. Lack of uptake by clinicians.	4 months to 3 years

We identified relevant literature through PubMed searches (limited to English-language publications) and author libraries. We included original and review articles and systematic reviews published between 2004 and 2020 to formulate an evidence-based narrative review focusing on interventions that have resulted in a decrease in antibiotic prescribing at the point of care in primary care and reflect the *core elements of outpatient antibiotic stewardship* (Table 1). Search terms ‘antimicrobial stewardship’, ‘outpatient’ and ‘primary care’ were used. For the purpose of this manuscript, primary care was defined as clinicians working in the community or ambulatory care settings, excluding sub-specialities. This narrative review may not capture all the available literature, however with experience and knowledge in this field we have attempted to include a relevant range of high quality publications and studies.

## AMS interventions in the primary care setting

Strategies from the framework should focus not only on appropriate use, but also on sustainability of behavioural change for both clinicians and patients [41]. Improving antibiotic prescribing at the point of care requires two complementary strategies [1]: changing clinician behaviour and alleviating concerns related to diagnostic uncertainties; and [2] educating patients and families about the role of antibiotics in medical care and their own wellbeing [42]. In addition, relationships should be fostered with potential partnerships and stakeholders such as national and local health departments, health plans and payers (health insurance companies), patients, community pharmacies and pharmacists, local microbiologic laboratories and professional organizations as part of a collaborative approach to optimise antibiotic use in the community [12].

### Commitment

Commitment as defined by the framework is the demonstration of dedication to and accountability for optimising antibiotic prescribing and patient safety. It is recommended to appoint a clinical leader who is accountable to senior facility leaders to promote appropriate antibiotic prescribing [14]. For an AMS program to be effective it is important to have the commitment of all staff at a practice level as well as to demonstrate the appropriate uptake of these interventions. Commitment to AMS should also be supported and strengthened by building AMS strategies into practice accreditation requirements as demonstrated in a Dutch trial [43].

An example of clinicians’ demonstration of commitment to appropriate antibiotic prescribing is by displaying a public statement in support of AMS in the clinic [14], which was associated with a 20% decrease in antibiotic prescribing in an randomized controlled trial [15]. A limitation of this study was that the participating clinics were self-selected and highly motivated to improve appropriate antibiotic prescribing which is unlikely to reflect the real world situation where there are many competing demands in a practice setting and AMS interventions may not receive a high priority. However, this commitment poster can be readily adopted by a busy clinic with minimal impact on workflow once it has been endorsed by the practice and its clinicians. It also supports a whole of practice approach which empowers all staff. Clinicians who have demonstrated ownership of the process are more likely to be committed to the appropriate use of antibiotics.

### Action for practice

Many interventions at the point of care have shown to be effective in decreasing antibiotic prescribing in primary care. These include a variety of strategies aimed at the clinician and/or patients. Using evidence-based diagnostic criteria and treatment recommendations such as clinical decision support, back-up (delayed or ‘wait and see’) prescribing and point of care tests are key strategies to change clinician behaviour. The debate is how these interventions can be implemented in a sustainable manner in the primary care setting.

#### Clinical decision support

Many clinical decision support tools have been developed and incorporated into clinical practice. These tools come in either printed materials or can be incorporated into electronic prescribing systems. Most of these interventions have been developed for the management of respiratory tract infections with variable effectiveness (Table 2).

**Table 2:** Examples of Clinical Decision Support Tools

Intervention	Impact on antibiotic use
Documenting clinical indications [16, 44]	Reduction in antibiotic prescribing.
Clinical prediction rules integrated into Electronic Health Records [18]	Reduction in antibiotic prescribing.
Clinical pathways/guidelines (print based or Electronic Health Record strategies) and patient education materials [17, 19, 32, 45, 46]	Reduction in antibiotic prescribing.
	Greater adherence to guidelines.
	Increase in appropriate antibiotic prescribing.
	Decrease in use of broad-spectrum antibiotics.

A number of these clinical decision support tools have been integrated into electronic prescribing with the support of an Electronic Health Record platform. These tools have been associated with reduced inappropriate antibiotic prescribing [16–18]; however, they have mainly been evaluated in study settings for limited time periods. Evaluation of the impact over a longer period is warranted to determine if these results can be sustained as well as providing reassurance that the outcomes are not due to diagnostic shifting in order to provide a systematic “gaming” of the process. For the successful implementation and uptake of these clinical decision support tools it is important that they promote appropriate prescribing of antibiotics according to evidence based guidelines and fit into the clinician’s workflow.

#### Back-up prescribing or watchful waiting

When there is clinical uncertainty about whether a condition is self-limiting or is likely to deteriorate, back-up prescribing (delayed prescribing) offers clinicians an alternative to immediate antimicrobial prescribing. It involves giving patients a prescription but suggesting that they not fill it unless they begin to feel worse or develop specific symptoms [47]. The first evidence of benefit from a randomised controlled trial of delayed prescribing came from Little et al in 1997 [20]. Several trials have been conducted since then [23, 24] and a Cochrane review has shown that ‘back-up prescribing’ results in a significant reduction in antibiotic use (32% of patients using antibiotics in the back-up prescribing group compared to 93% of patients in the immediate prescription group) [21]. A limitation of delayed antibiotic prescribing is that it can perpetuate the notion that antibiotics are the solution for common self-limiting infections in the community [48]. In addition, delayed antibiotic prescriptions also allows for the inappropriate use of antibiotics when patients fill these prescriptions for conditions other than the initial diagnosis.

An alternative strategy to delayed antibiotic prescribing is the watchful waiting approach which involves providing symptomatic relief with a clear plan for follow-up if symptoms worsen or do not improve. It has been shown to safely decrease antibiotic use when used in accordance with clinical practice guidelines [22]. This strategy has been included in national guidelines [47, 49, 50]. For instance, it is recommended by the American Academy of Paediatrics’ and in the Australian *Therapeutic Guidelines: antibiotic* in the management of acute otitis media and sinusitis [49–51].

#### Point of care patient tests

Two types of point of care (POC) tests exist to support clinical decision making in infections; 1) tests measuring the level of non-specific inflammatory markers in the blood (such as CRP) and 2) tests assessing the presence of a pathogen (such as the Rapid Streptococcal Antigen Detection test) [29].

The CRP test is widely used in primary care in some European countries [25]. A Cochrane review concluded that CRP used as an adjunct to a doctor’s clinical examination can reduce antibiotic use for patients with ARIs in the primary care setting (risk ratio (RR) 0.78, 95% confidence interval (CI) 0.66 to 0.92. Do and colleagues supported these findings which found C-reactive protein was effective in reducing antibiotic use with no apparent risk to patient safety [28].

For nearly 30 years, the rapid antigen detection test (RADT) has been available for detection of Lancefield group A β-hemolytic streptococci (GABHS) in patients with a sore throat. In Europe, there is a lack of consensus for the use of RADT. A European trial has demonstrated there is no additive effect of utilising RADT in patients with a sore throat either for symptomatic or antibiotic management as compared to a clinical score alone to guide antibiotic prescribing [29]. The United States of America’s guidelines recommend testing for the Group A Streptococcal (GAS) pharyngitis utilising the RADT and/or culture because clinical features alone do not reliably discriminate between GAS and viral pharyngitis. In addition, the generally high specificity of RADT should minimize over-prescription of antimicrobials for treatment of adult patient with GAS pharyngitis [30]. Despite the accuracy of the test clinicians still tend to rely more on their clinical judgment which highlights the importance of education and addressing behavioural issues relating to the interpretation of the test.

Point of care tests have the potential to decrease diagnostic uncertainty, support non-prescription decisions, and to reassure patients [30]. However, concerns expressed by clinicians relating to accuracy, misleading results, and over-reliance on diagnostic tests need to be addressed [52]. Point-of-care tests are likely to be more effective if they can be integrated into existing practice workflow. For example, tests that require a patient to wait for results in the office beyond the scheduled appointment time or require a clinician to call later with results are likely to have lower uptake. Another limitation is that in many countries point of care tests are not reimbursable.

### Tracking and reporting (audit and feedback)

Tracking and reporting, which is reviewing clinician antibiotic prescribing and providing direct feedback to the prescriber, has been shown to reduce antibiotic prescribing [14, 16, 19, 32–34, 53]. Meeker et al. showed that use of ‘accountable justification’ (prompting clinicians to justify why they prescribed an antibiotic) and peer comparison as behavioural interventions can resulted in a reduction in inappropriate antibiotic prescribing for acute respiratory tract infections [16]. A study from the United Kingdom evaluated a large scale feedback intervention where high prescribing practices received a personalised letter from England’s Chief Medical Officer stating that they where prescribing antibiotics at a higher rate than 80% of practices in their National Health Service Local Area Team. This simple intervention resulted in a 3.3% reduction of dispensed antibiotics over 6 months [31]. A similar intervention was trialled in Australia where a personalised letter containing a peer comparison graph resulted in a reduction in antibiotic prescribing rates of 12.3% over the six-month period. This effect was more pronounced than other interventions such as an education-only letter about antibiotic prescribing which reduced antibiotic prescriptions by 3.2% [54]. In order for an intervention such as this to succeed it is important that feedback to prescribers is provided through a person of authority, such as a Chief Medical Officer. In a study conducted in Switzerland where primary care physicians with the highest antibiotic prescription rates were given feedback by a company owned by health insurers, antibiotic prescribing was not associated with a change of antibiotic use [55]. These peer comparison feedback tools where feedback has been provided by a respected figure of authority have demonstrated an ability to reduce antibiotic prescribing at low cost and on a national scale, which makes them a worthwhile addition to AMS programs.

Public reporting of antibiotic usage data across medical practices and individual providers to enable national benchmarking and possibly reimbursement modification is another strategy that is being explored in some countries in order to promote appropriate use of antibiotics [35].

It is important to note that uptake of AMS interventions does not happen spontaneously; rather an active implementation approach over the long term is required [56]. Gerber et al was able to show that a combination of clinician-specific education and audit and feedback significantly reduced prescribing of antibiotics in the primary care setting [53]. Following the removal of audit and feedback, however, the initial benefits of this primary care antimicrobial stewardship intervention diminished over time [57]. This highlights that these interventions should be implemented for the long-term in order for the effects to be sustainable [58].

Although a number of benefits have been demonstrated with audit and feedback to prescribers it is not without its challenges. The auditing process relies on practices having an Electronic Health Record system in place in order for the data to be extracted. If more in depth audits are being conducted, such as evaluating adherence to guidelines, then an important factor is the accurate documentation of patient information, for instance an indication for the antibiotic is readily accessible in the medical records. The auditing process is also time consuming and requires additional resources. In addition, the auditors need to be trained and have the expertise to validate and interpret the data.

### Education

Providing educational resources on antibiotic prescribing to clinicians and patients, and ensuring access to relevant expertise on optimising antibiotic prescribing is a core element of the CDC framework [14]. It is important that clinicians are providing relevant evidence based education to their patients in order to ensure that there is a consistent message to consumers about appropriate use of antibiotics. These educational activities should be tailored to clinicians and patients taking into consideration local beliefs and cultures.

It is important to be aware that outpatient antibiotic prescribing is also driven by psycho-social factors, including lack of self-awareness, fear of complications and perceived patient expectations [59]. Patient communication strategies should include a combination of evidence based practice with effective patient communication in order to address these factors [36]. A study by Mangione-Smith et al. showed that communication strategies that provided parents of children presenting with viral URTIs with positive treatment recommendations to reduce their child’s symptoms decreased antibiotic prescribing. A combination of positive and negative recommendations (what not to do) also reduced antibiotic use while maintaining high patient satisfaction [60].

Strategies such as shared decision making, have been utilized to educate patients about when antibiotics are and are not needed. Shared decision making tools have been shown to reduce antibiotic prescribing for ARIs from 47 to 29% as compared to standard management (RR 0.61 95% CI 0.55 to 0.68) without an increase of patient-initiated re-consultations (RR 0.87; 95% CI (0.74 to 1.03) and also without impacting on patient satisfaction (OR 0.86; 95% CI (0.57 to 1.30) [37].

There are a number of education activities targeting clinicians. Didactic training for clinicians is only marginally effective whereas the greatest effect has been shown when it is accompanied by communication skills training and support [26, 33, 38–40]. Primary care clinicians who have been trained in enhanced communication skills have demonstrated long term benefits, prescribing significantly fewer antibiotics [38]. In addition, these interventions have also demonstrated they increase clinicians’ knowledge and confidence in diagnosing an infection [61] whilst helping maintain parental visit satisfaction [60].

## Discussion

This paper proposes that in order to facilitate the change management process for appropriate use of antibiotics in the primary care setting, a top-down approach is needed with a combination of regulatory and policy decision making processes in place at a national level supported by the framework addressing the *Core Elements of Outpatient Antibiotic Stewardship*. This should be complemented by a bottom-up approach utilising practice level AMS interventions that can be scalable to a national level. The question is how this can be achieved in the community where in many cases care is delivered in small scale clinics. We propose a tiered approach to support the implementation of AMS strategies in the clinical practice setting [62] which includes resources to fund the implementation of these interventions that are connected to quality improvement initiatives, as well as collaboration with partners and key stakeholders and the implementation of national accreditation standards.

Implementation of AMS models requires both the leadership and culture change supported by policy development. External motivators such as embedding AMS frameworks such as the *Core Elements of Outpatient Antibiotic Stewardship* into national accreditation standards should also be explored [52]. Accreditation standards in the hospital setting have shown to be a main driver in the implementation of AMS programs as well as funding the positions that support these programs [11]; however, in countries such as Australia AMS activities are merely an option to be considered in the accreditation process for out of hospital care [63]. In the United States if sites are accredited by The Joint Commission, then as of 2020 there will be a requirement for Joint Commission-accredited ambulatory health care organizations that routinely prescribe antimicrobial medications to meet the new antimicrobial stewardship requirements. However, the majority of outpatient practices are not accredited by The Joint Commission. When compared with benchmark countries, antimicrobial dispensing rates in Australia are substantially higher than Sweden, where a national AMS strategy has successfully been implemented over a long period of time [2].

This paper highlights that the interventions described above have mainly being conducted in a research setting with limited length of observations. Interventions utilised at the point of care such as those focusing on patient doctor interactions and knowledge of antibiotics have been associated with increased adherence and acceptance of treatment and often led to lower rates of antibiotics [64]. In order to optimise the uptake of these interventions they should be supported with strategies such as education and training, decision support systems and audit and feedback [65]. However, even using a sustained multifaceted community-level intervention over three years has only been modestly successful at decreasing overall antibiotic use beyond the secular trends in the community [15]. In addition, how sustainable and generalizable these interventions are beyond the research setting needs to be determined. Novel approaches will be needed to further reduce in appropriate antibiotic rates such as incorporating incentives into daily practice. Although new approaches may emerge from further primary research into understanding the psychology that underlies inappropriate prescriptions, policies that align the economics of self-interest with societal need and quality improvement initiatives, are most likely to result in sustained improvements [35].

Incentives that have been identified for implementing AMS strategies in primary care include the need for a funding model and reimbursement strategy that supports AMS activities and penalises clinicians and practices that fail to adopt AMS. These facilitators likely require governmental policy change, although they could also be considered by third party payers [52]. Countries such as Sweden and United Kingdom have a more centralized health care system which lends itself to a national AMS strategy in the primary care setting. In the UK the introduction of financial incentives for local commissioners of healthcare to improve the quality of prescribing was associated with a reduction in antibiotic prescribing in primary care [66]. In other countries, with a more privatised health care system and a variety of reimbursement strategies, implementation of AMS strategies are more challenging and the focus may need to be at a local rather than a national level.

A few countries have implemented AMS strategies in primary care with various degrees of success. For instance the successful Swedish strategy known as Strama (Swedish strategic program against antibiotic resistance) was created in 1995 and started off with a bottom-up approach of coordinating activities for the containment of antibiotic resistance in the community. In order to support clinicians in the community, a top-down strategy was added including setting a national target for the number of prescriptions in outpatient care and defining quality indicators based on treatment recommendations, as well as providing local feedback to prescribers. It is important to note that sustainable funding was allocated for these strategies at both a national and local level [67]. In January 2019 the United Kingdom published a cross-government national strategy to tackle antimicrobial resistance utilising a One Health Approach to be implemented in all practice settings. It also includes predefined targets in reducing antimicrobial use and drug resistant infections [68]. This was accompanied by the UK’s vision for AMR in 2040 which has been defined as ‘a world where AMR is controlled and contained through mitigation’. [69] The current strategy in UK primary care utilises an antibiotic toolkit named TARGET (Treat Antibiotics Responsibly, Guidance, Education, Tools) which provides diverse resources to support health professionals and educate patients in appropriate use of antibiotics. It has been well received [13], and has contributed to a reduction of 7.3% in antibiotic DDDs/1000 inhabitants/day in the last four years [69].

Successful AMS adoption models in European countries showed a distinct pattern: a slow and long early adoption phase at the practice level followed by policy changes that eventually trigger large-scale adoption. In addition, a few opinion leaders became early adopters, recognising the importance of using AMS strategies to support their decision-making in antibiotic prescriptions [52].

The framework *Core Elements of Outpatient Antibiotic Stewardship* provides the ideal platform to support the successful implementation and uptake of AMS programs in primary care because it utilises the combination of education, guidance, interventions and a supportive policy environment. It is also important to remember that for an AMS program to be effective it requires the commitment of all staff at a practice level in order to ensure the uptake of these interventions. Clinicians at the point of care are well placed to be effective stewards of antimicrobials and every clinician is ultimately responsible for appropriate use of antibiotics.

In summary, in order to ensure the sustainable implementation and uptake of AMS interventions in the community a top down approach complementing a bottom-up commitment and utilising a combination of strategies is warranted. National accreditation standards are essential and could be linked to the framework *Core Elements of Outpatient Antibiotic Stewardship.* Sufficient resources should support such a strategy in order to ensure the implementation of interventions connected to quality improvement initiatives. Without these strategies the problem of inappropriate antibiotic prescribing in the community will not be adequately addressed and the successful implementation and uptake of AMS programs will remain a dream.

## Data Availability

Not applicable.
